# Synthesis of potent MDA-MB 231 breast cancer drug molecules from single step

**DOI:** 10.1038/s41598-023-45455-y

**Published:** 2023-10-25

**Authors:** Senthilnathan Govindaraj, Kilivelu Ganesan, Mahendiran Dharmasivam, Lakshmisundaram Raman, Mohammed Mujahid Alam, Mohammed Amanullah

**Affiliations:** 1 PG& Research Department of Chemistry, Presidency College, Chennai, 600005 India; 2 Centre for Cancer Cell Biology and Drug Discovery, Griffith Institute for Drug Discovery, Griffith University, Nathan, Brisbane, QLD 4111 Australia; 3 Sri Ramachandra Faculty of Pharmacy, Sri Ramachandra Institute of Higher Educational and Research (DU), Porur, Chennai, 600116 India; 4 Department of Chemistry, College of Science, King Khalid University, PO Box 9004, Abha, 61413 Kingdom of Saudi Arabia; 5 Department of Clinical Biochemistry, College of Medicine, King Khalid University, Abha, 61413 Kingdom of Saudi Arabia

**Keywords:** Biochemistry, Cancer, Drug discovery, Molecular biology, Oncology, Chemistry

## Abstract

We have prepared novel potent breast cancer drug molecules from non-toxic and inexpensive method. Column chromatography is not necessary for purification of target molecules. The value of overall atom economy, environmental factor, environmental catalyst and product mass intensity gives additional merits for this synthetic method. Synthesized flexible dimeric imidazolium bromides showed less toxicity and gives excellent anticancer response against normal mammary epithelial cells. Novel dimeric pyridinium bromides showed excellent anticancer response against tested cancer cell lines. In cell cycle, novel flexible dimeric pyridinium bromides showed significant arrest in the G2/M phase by nearly three folds, when compared with control drug. We have studied the targeting epidermal growth factor receptor for all the synthesized flexible amino substituted and methyl substituted dimeric pyridinium bromides.

## Introduction

Organic cation binds with inorganic anion (or) Inorganic cation binds with organic anion (or) both cation and anion from organic moieties which are in liquid nature at room temperature are called room temperature ionic liquids (RTIL). In the structure description of ionic liquids, it is an electrically neutral molten salt consisting of equal number of cation and anion in the liquid form. RTIL shows, unique thermo physical characters such as low melting salt, highly viscous in nature, thermally more stable and have greater electrochemical properties^1^. Organic cation like imidazolium, pyridinium and pyrazolium ion with simple inorganic anions are mostly used in pharma industries due to its unique physiochemical behaviors^2,3^. The quarternization of sp^2^ nitrogen of pyridine and imidazole nucleus is the important method in the synthesis of ionic liquids^4–10^. Pyridine derivatives are very important and useful moieties to reach wide popularity in the Bronsted acidic ionic liquids to prepare the drug molecules^11–18^. Binary mixture are prepared from 1-butuyl-3-methyl imidazolium cation with acetate anion and Doxorubicin or Mitoxantrone and its cytotoxicity is studied against human colorectal adenocarcinoma CaCo-2 cells and showed higher cytotoxicity than the Colo 320 HSRcells^19,20^. Monomeric imidazolium cation based ionic liquids showed excellent anti-tumor activities^21^. Alkyl chain length from C_2_–C_8_ substituted imidazolium bromide/hydroxide type of ionic liquids showed moderate to significant vermicidal response^22^. Water soluble betulinic acid counter anion with trihexyl tetradecyl phosphonium cation type of ionic liquids are acted as selective antitumor agent against Hep G2 (liver), MG63 (Osteosarcoma), T47 D (breast), A459(lung) and RKO (colon) cancer cells^23^. Electron donating substituted benzofused phosphorlactones showed potential anti-pancreatic cancer cell which was identified by cell cycle experiment^24^. Ammonium and phosphonium cation with simple and alkyl substituted anion showed better cytotoxicity response against leukemia cell lines. Higher alkyl chain containing phosphonium type of ionic liquids showed excellent anti-cancer activity against breast cancer and ovarian cancer cells with GI50 values^25^. Saeid et al. reported that, the anti-cancer properties of lipid nano encapsulated and starch nano-encapsulated form of the Samarium complex was studied against cell lines of A-549 and MCF-7 by MTT method. Lipid and starch nano-encapsulated molecules showed higher antitumor and antimicrobial response than the Samarium complex alone^26^. Recent researchers are focusing their finding towards the preparation and identification the non-toxic anti-cancer drug molecule which is not yet available^27–32^. We wish to prepare cheaper and water soluble anticancer drug molecules with lipophilic and hydrophilic moieties from substituted pyridinium salts under shortest synthetic methodology; we hope that, will get good response in the study of anti-cancer properties of substituted pyridinium salts against breast cancer cell line under MTT assay.

## Results and discussion

**Figure 1:**
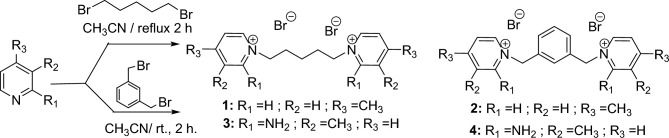
Synthesis of flexible substituted dimeric pyridinium bromides.

Synthesis of some novel dimeric pyridinium bromides **1–4** from easily available and cheapest starting materials under simple synthetic procedure. 1,5-Dibromopentane/*m*-xylene dibromide and 2.02 equivalents of 4-methlpyridine/2-amino-3-methylpyridine are dissolved in CH_3_CN followed by refluxtion for about2-3 h afforded the quarternized flexible dimeric pyridinium bromide **1–4** in 92–95% yield (Fig. 1). Formation of aryl moiety linked dimeric pyridinium bromide **2**, **4** is much faster than the flexible alkyl moiety linked dimeric pyridinium bromides **1**, **3** due to *m*-xylene dibromide which reacts much facile than the 1,5-dibromopentanebecause of less inductive effect. The schematic reaction scheme as follows.

*Atom Economy (AE)* Atom economy of 1, 5-dibromo pentanereacts with 4-methyl pyridine is defined as how much reactant are converted into dimeric pyridinium bromide **1**.

**Figure 2:**
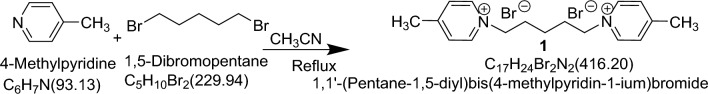
Chemical reaction used for atom economy calculation.

The atom economy is calculated for the following reaction (Fig. 2).

$${\text{Atom}}\;{\text{ economy }} = {\text{ Molecular }}\;{\text{weight }}\;{\text{of}}\;{\text{ product/}}\sum \left( {{\text{Molecular}}\;{\text{ weight }}\;{\text{of }}\;{\text{reactant}}} \right) \times {1}00$$

$${\text{AE}} = { 416}.{2}0\;{\text{g}}/{\text{mol/}} \left( {{93}.{13} + { 93}.{13} + { 229}.{94}} \right) \times {1}00$$

$${\text{AE }} = {1}00 \, \%$$

*Environmental factor (EF)* The environmental factor is calculated for the following reaction by ratio of mass value of unwanted (or) wasted materials/Mass value of product molecule.

$${\text{Environment}}\;{\text{ factor }} = {\text{ Mass }}\;{\text{value}}\;{\text{ of }}\;{\text{waste }}/{\text{ Mass }}\;{\text{value }}\;{\text{of }}\;{\text{product}}$$

**Figure 3:**
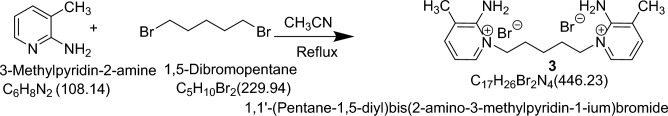
Chemical reaction used for environmental factor calculation.

Mass of waste is calculated as follow (Fig. 3):

$$\begin{aligned} {\text{Mass}}\;{\text{ of }}\;{\text{waste}} & = {\text{ Net }}\;{\text{mass }}\;{\text{of }}\;{\text{starting }}\;{\text{materials }}{-}{\text{ Net }}\;{\text{mass }}\;{\text{of }}\;{\text{product}}\;{\text{ obtained}} \\ & = \left( {{229}.{95}\;{\text{g}}/{\text{mol}} + { 1}0{8}.{14}\;{\text{g}}/{\text{mol}} + { 1}0{8}.{14}\;{\text{g}}/{\text{mol}}} \right){446}.{23}\;{\text{ g}}/{\text{mol}} \\ & = {446}.{23 } - { 446}.{23} \\ \end{aligned}$$

$${\text{Environment factor }} = \, 0$$

So,

$${\text{E - factor }} = {\text{ Mass}}\;{\text{ of}}\;{\text{ waste }}/{\text{ Mass }}\;{\text{of}}\;{\text{ product}}$$

$${\text{E - factor }} = 0$$

$${\text{E - factor }} = 0 \, \left( {{\text{Ideal }}\;{\text{value}}\;{\text{ of }}\;{\text{E - factor }}\;{\text{is }}\;{\text{considered }}\;{\text{zero}}} \right)$$

*Product mass intensity (PMI)* The ratio between total mass of (reactant + solvent) and mass of expected product.

**Figure 4:**
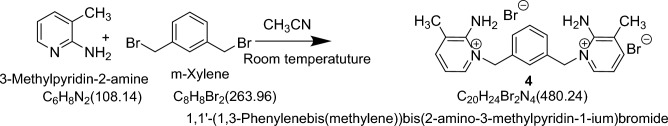
Chemical reaction used for product mass intensity calculation.

We have calculated product mass intensity of the above reaction by using following formula (Fig. 4)

$${\text{PMI}} = \, \sum ({\text{Mass }}\;{\text{of }}\;{\text{reactants}} + {\text{Solvent}}) \, /{\text{ Mass }}\;{\text{of}}\;{\text{ product}}$$

$$\begin{aligned} {\text{PMI}} = & \, \left( {{263}.{96} + {1}0{8}.{14} + {1}0{8}.{14} + {41}.{1}} \right)/{48}0.{24} \\ = & { 521}.{34}/{48}0.{24} \\ = & {1}.0{8} \\ \end{aligned}$$

Similarly, we have calculated the atom economy, environmental factors and product mass intensity of remaining reactions and presented in the Table 1. We have used non-toxic, low boiling solvent; mass value is given in the Table 1 which is used for all reaction. We have not used any expensive, toxic and environmental hazardous catalyst for the preparation of our target drug molecules.

**Table 1:** Atom economy, environmental factors, product mass intensity for flexible substituted dimeric pyridinium bromides.

Compd	No. of steps	Overall yield (%)	Overall AE (%)	E-factor	E-solvent	E-catalyst	Product mass intensity (PMI) total
**1**	1	2.94	100	0	41.01	0	1.09
**2**	1	3.78	100	0	41.01	0	1.09
**3**	1	3.79	100	0	41.01	0	1.09
**4**	1	3.46	100	0	41.01	0	1.08

## Biological studies

### Anticancer activity

#### MTT assay

Ionic liquids have rarely or never been tested for anticancer studies and our previous ionic liquid compounds showed potent anticancer activity^33^. Therefore, it is very important to evaluate the anticancer activity of newly discovered compounds against various cancer cells.

IC_50_ values are greater than 20 for MCF-7 and MDA-MB-231 breast cancer cell lines, IC_50_ values greater than 30 for MCF-10A breast cancer cell lines in 24 h incubation period with reference to doxorubicin which is used as a reference drug molecules^34^. IC_50_ values for *Phyllanthus niuri* DCM extract against MCF-7 and MCF-7 ADR breast cancer cell lines showed 100 in 24 h incubation period^35^. Sumit et al. prepared from triazole tethered Ospemifene-isatin conjugated drug molecules using expensive catalyst for McMurry coupling followed by additional four steps to get their triazole tethered Ospemifene-isatin conjugated drug molecules. IC_50_ values of different alkyl chain length containing triazole tethered ospemifene-isatin derivatives are 53.10 and 70.71 for two and three methylene linkers respectively^36^. Dibiotin ester linked Ruthenium complex and *cis*-platin showed IC_50_ values of 31.5 ± 4.7, 38 ± 1.41 against MCF 7 breast cancer cell line in 24 h incubation^37^. Mina and coworker reported the breast cancer effect of Metformin drug molecules on T47D breast cancer cell lines. IC_50_ value for Metformin and Silibinin drug molecules are 21.20, 106.50 respectively in 24 h incubation period against T47D breast cancer cell lines^38^.

**Figure 5:**
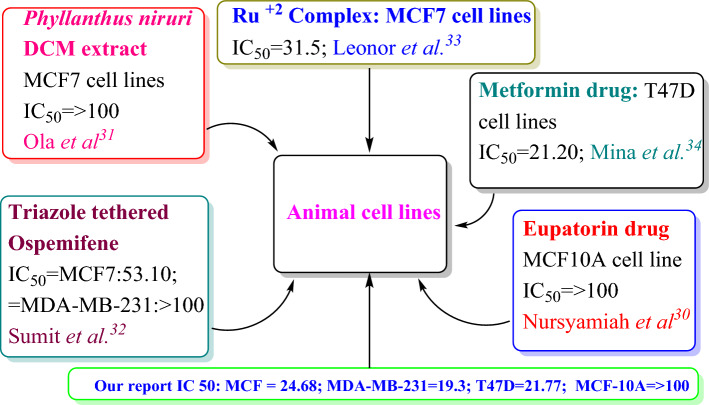
IC_50_ values comparison between available literature and our target molecules.

The anticancer activity of the compounds (**1–4**) was tested against three cancer cell lines namely human breast adenocarcinoma (MCF-7), (MDA-MB-231), and ductal carcinoma (T47D), and one human mammary epithelial (MCF-10A) cell line by MTT assay. The investigated anticancer activity of the compounds was time-dependent, i.e., cell viability decreased with increasing incubation, and their IC_50_ values are listed in Table 2. IC_50_ values of the compounds showed less toxicity against normal human mammary epithelial cells and more toxicity against cancer cells. All the compounds have efficiently killed the MDA-MB-231 cancer cell line compared to other tested cells (Fig. 5). Compound **1** and **2** demonstrated remarkable anticancer activity compared to other compounds against all the tested cell lines. Interestingly, the presence of an electron-donating methyl substituent in compound **2** increases lipophilicity and allows it to easily cross the blood–brain barrier and kill all cancer cells.

**Table 2:** IC_50_ values of compounds against MCF-7, MDA-MB-231, TD47D and MCF-10A cell lines.

Dimeric pyridinium bromide (DPB)	IC_50_ (µM)							
	MCF-7		MDA-MB-231		T47D		MCF-10A	
	24 h	72 h	24 h	72 h	24 h	72 h	24 h	72 h
DPB**1**	164.97 ± 0.21	29.04 ± 0.01	112.24 ± 0.54	23.64 ± 0.04	126.87 ± 0.41	25.92 ± 0.27	> 100	> 100
DPB**2**	158.07 ± 0.47	24.68 ± 0.35	105.67 ± 0.87	19.32 ± 0.21	113.21 ± 0.07	21.77 ± 0.11	> 100	> 100
DPB**3**	256.21 ± 0.02	47.23 ± 0.54	146.32 ± 0.11	36.89 ± 0.15	161.87 ± 0.52	39.65 ± 0.12	> 100	> 100
DPB**4**	189.69 ± 0.37	43.88 ± 0.18	138.87 ± 0.71	32.05 ± 0.02	157.38 ± 0.34	34.38 ± 0.01	> 100	> 100

#### Apoptosis

**Figure 6:**
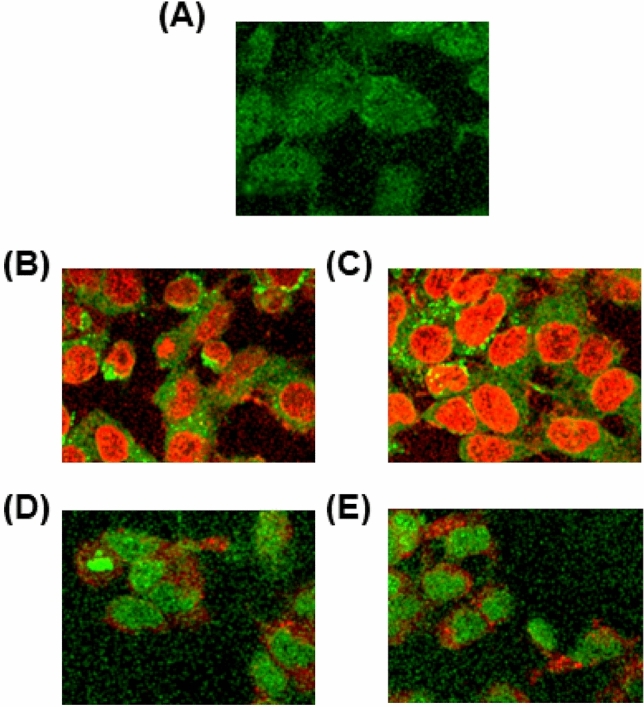
AO/EB assay of MDA-MB-231 cells after 24 h: (**A**) Control; (**B**) **1**; (**C**) **2**; (**D**) **3**; (**E**) **4**.

**Figure 7:**
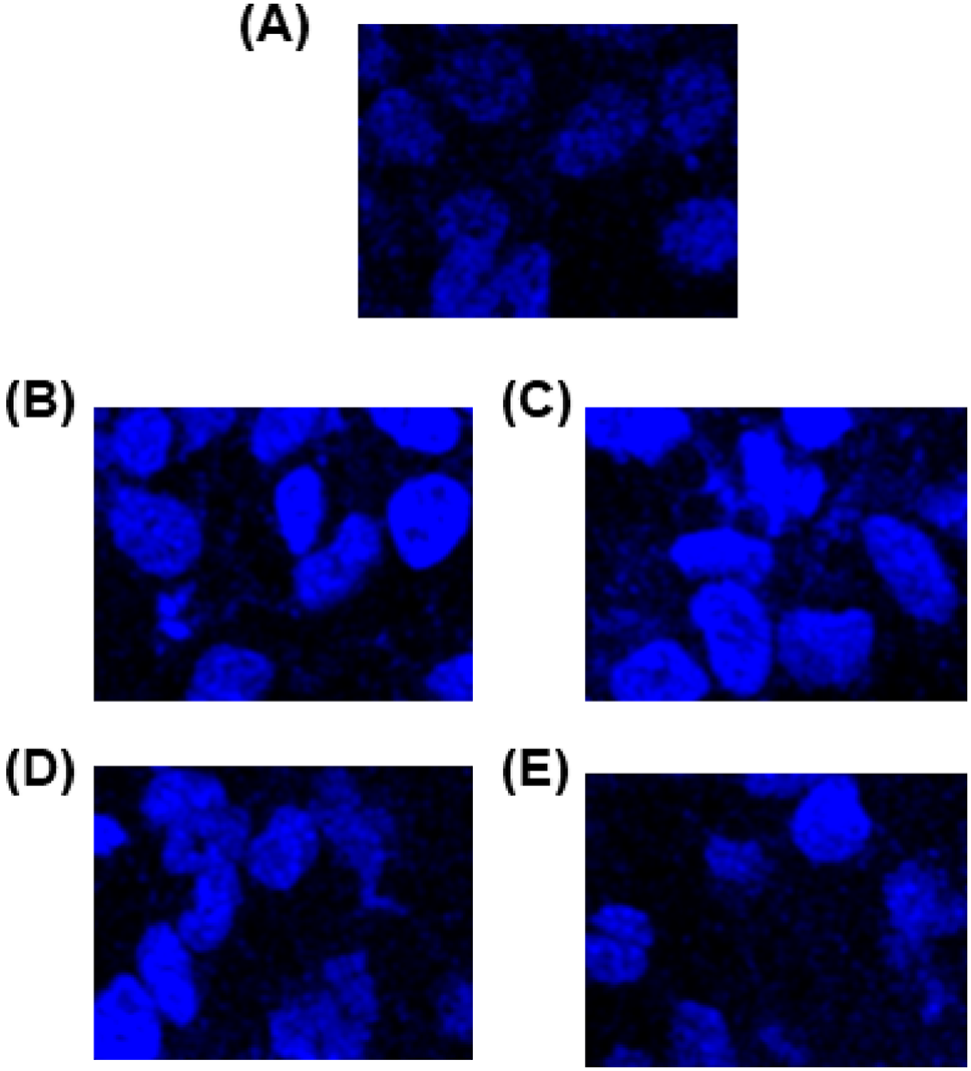
Hoechst 33258 assay of MDA-MB-231 cells after 24 h: (**A**) Control; (**B**) **1**; (**C**) **2**; (**D**) **3**; (**E**) **4**.

Many researchers have demonstrated that many anticancer drugs induce apoptosis, which plays an important role in cancer drug development^33^. For this reason, we tested the morphological changes of our new compounds against MDA-MB-231 cells with acridine orange (AO) and ethidium bromide (EB) after 24 h. Morphological assessment demonstrates viable cells (control) with normal morphology and pale green nuclei. However, the MDA-MB-231 cells treated with compounds for 24 h confirmed morphological changes as a greater part of cells become shrinkage, and green apoptotic cells containing apoptotic bodies and red necrotic cells were observed (Fig. 6). Further confirming the induction of apoptosis, the MDA-MB-231 cells were also treated with compounds and stained with Hoechst 33258 for 24 h, which exhibit apoptotic highlights like chromatin fragmentation, cytoplasmic vacuolation, nuclear swelling, and cytoplasmic blebbing (Fig. 7). These data strongly suggested these compounds induced apoptosis in MDA-MB-231 cells.

#### Cell cycle

**Figure 8:**
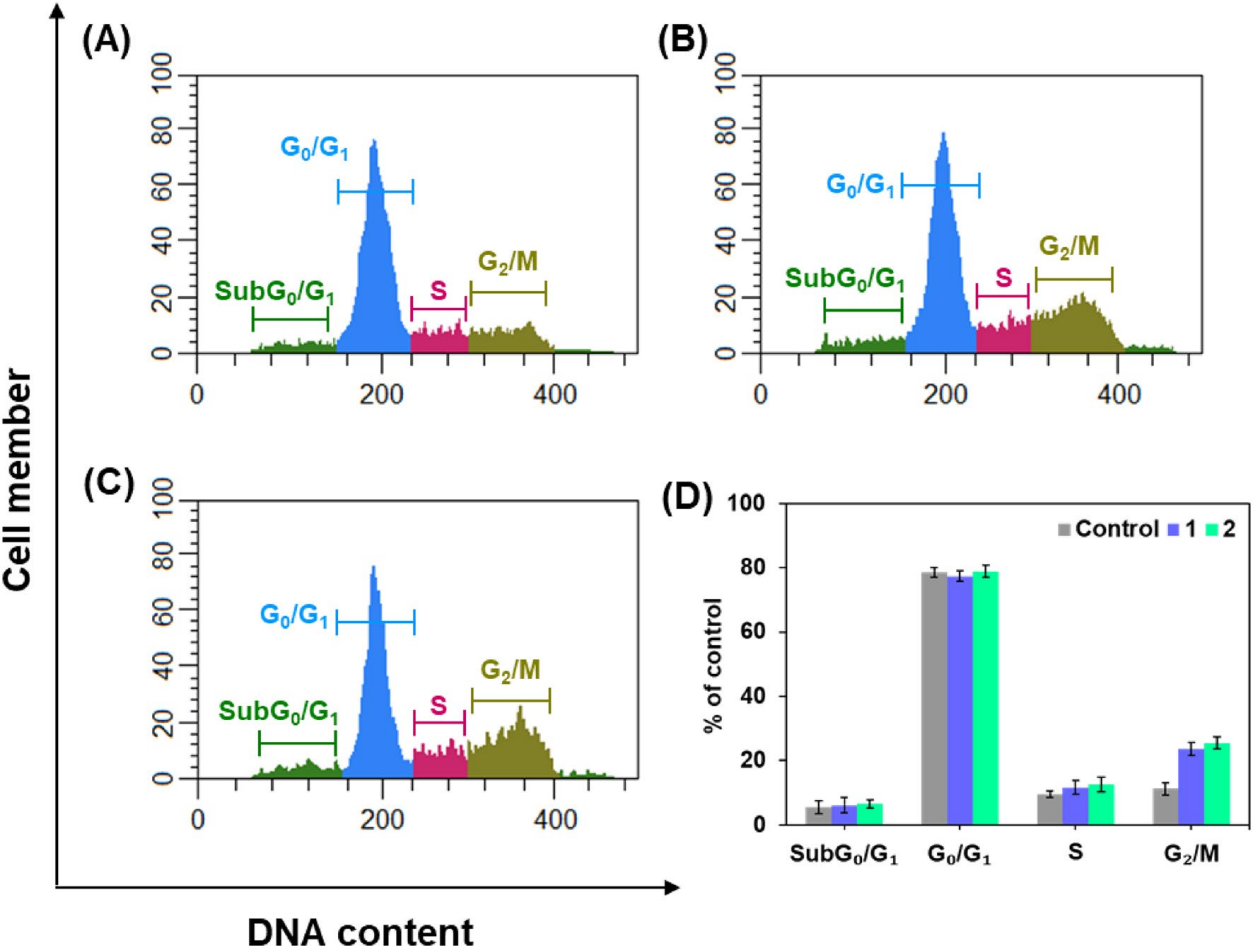
Cell cycle progression of MDA-MB-231 cells for 24 h: (**A**) Control; (**B**) **1**; (**C**) **2**; (**D**) Cell population.

Cell cycle analysis was also performed to further confirm that the newly discovered compounds showed good anticancer activity against MDA-MB 231. Therefore, MDA-MB 231 cells were treated with Alkyl/aryl linker unit containing flexible methyl substituted dimeric pyridinium bromides **1** and **2** for 24 h and their cell cycle profile and induction of apoptosis were analyzed. From (Fig. 8), it is demonstrated that the compounds did not induce apoptosis as no changes occurred in SubG_0_/G_1_. Figure 7A–E also show that the compounds did not cause any change in G0/G1and S-phase compared to the control. Interestingly, both compounds significantly arrested the G2/M phase by 2.5-fold compared to the control. Accumulation of cells in G2/M phases is a significant indication of the apoptotic role of alkyl/aryl linker unit containing flexible methyl substituted dimeric pyridinium bromides **1** and **2** in MDA-MB-231 cells.

### Induction of cell apoptosis with annexin V-FITC and propidium iodide (PI)

**Figure 9:**
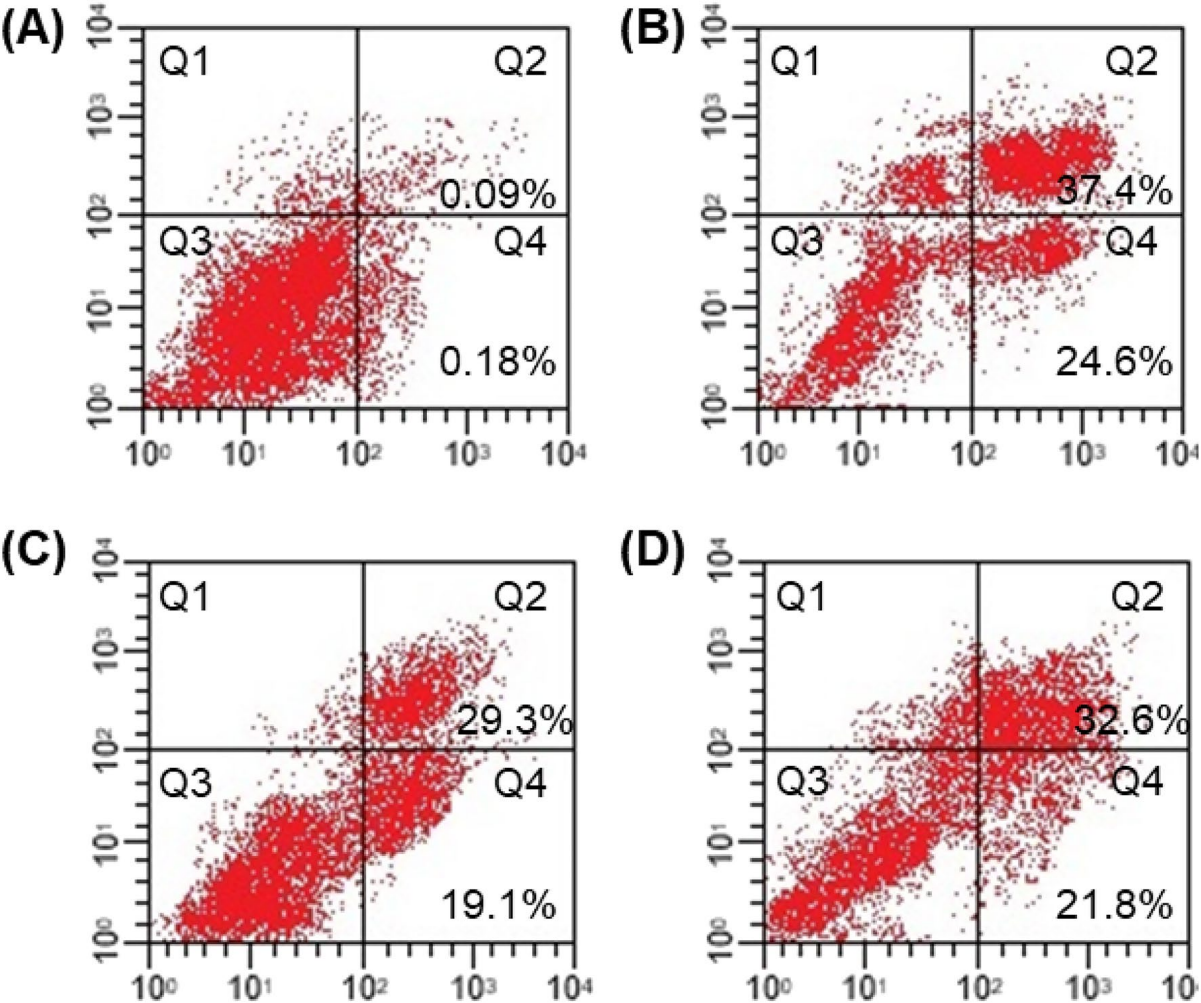
The assessment of apoptosis in MDA-MB-231 cells via the annexin V-FITC and propidium iodide assay at 48 h. The figure presents four separate conditions: (**A**) Control, (**B**) Positive control 5-fluorouracil, (**C**) Compound **1**, (**D**) Compound **2**. The figure show four distinct cell states within these conditions: necrotic cells (Q1), late apoptotic cells (Q2), viable cells (Q3), and early apoptotic cells (Q4). This method uses the protocol previously published in another study^39^.

The study aimed to investigate the impact of lead compounds **1** and **2**, along with the positive control 5-fluorouracil (25 μM), on MDA-MB-231 cell apoptosis and necrosis. MDA-MB-231 cells were exposed to the compounds for 48 h. Following treatment, cell nuclei were stained with annexin V-FITC and propidium iodide (PI) and analyzed through flow cytometry. The findings revealed significant alterations in the apoptotic and necrotic cell populations. In the absence of treatment, only 0.09% of the cells exhibited late apoptotic characteristics, as indicated by their placement in quadrant Q2. However, when exposed to 25 μM of compounds **1** and **2**, the late apoptotic cell population increased to 29.3% and 21.8%, respectively (Fig. 9). It is noteworthy that both compounds 1 and 2 exhibited lower activities while compared to the positive control, 5-fluorouracil (37.4%).

In the case of early apoptosis (quadrant Q4), 0.18% of the control cells displayed such features. This proportion significantly increased to 19.1% and 21.8% for compounds 1 and 2, respectively, post-treatment (Fig. 9). Once again, compounds **1** and **2** were less effective than 5-fluorouracil (24.6%) in inducing early apoptosis. Interestingly, compound **2** also induced necrotic cell death relative to the control (Fig. 9). The observed changes in apoptosis and necrosis were substantiated by additional staining methods, including AO/EB and Hoechst 33,258, further supported by flow cytometry analysis.

### In silico pharmacokinetics

**Figure 10:**
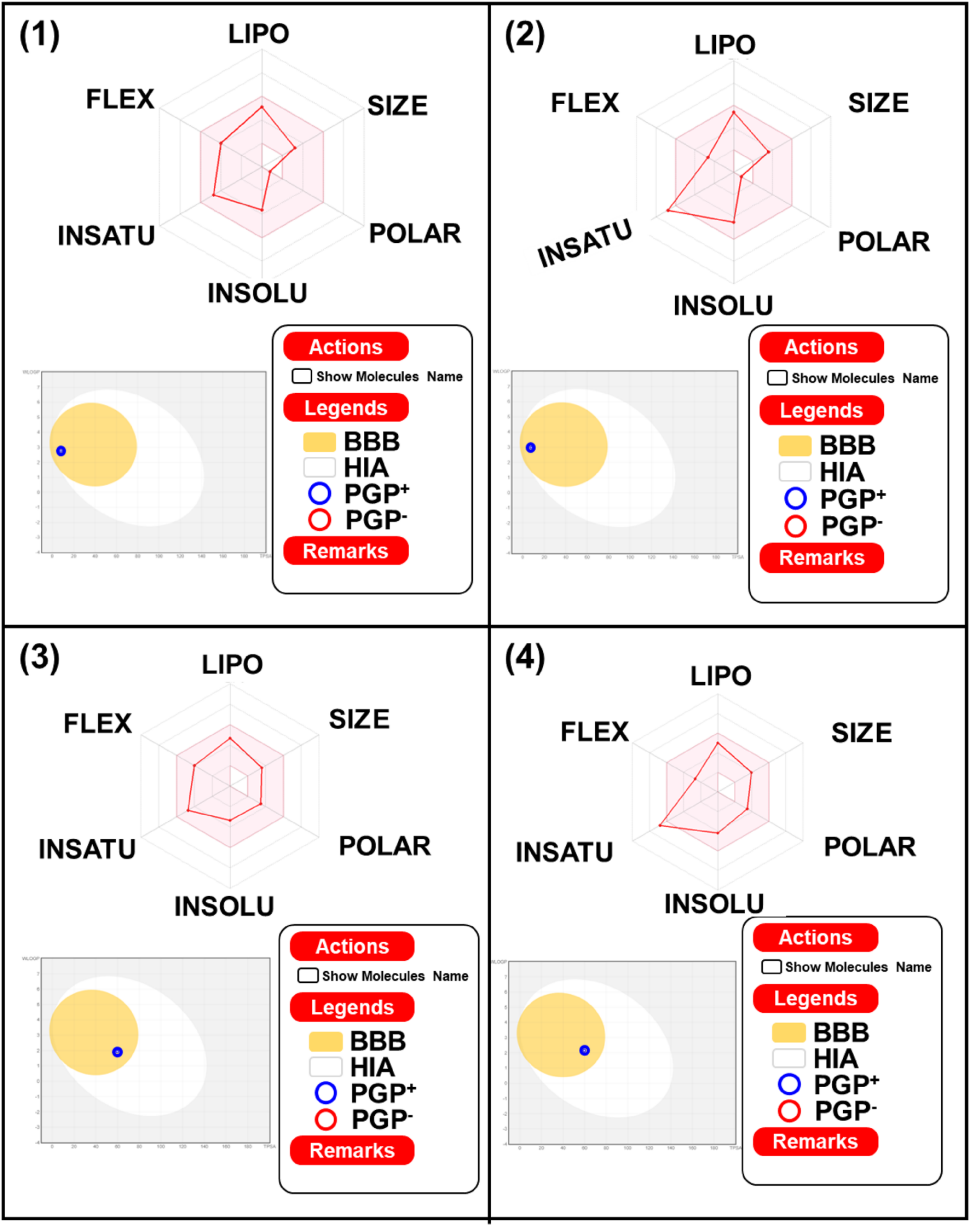
Drug likeness parameters of dimeric pyridinium bromides **1**–**4** assessed using Swiss ADME. *LIPO* lipophilicity, *POLAR* polarity, *FLEX* flexibility, *INSATU* insaturation or saturation asper the fraction of carbons in sp3 hybridization, *INSOLU* solubility.

Swiss drug design tools were used to predict the bioavailability of compounds (**1**–**4**)^33^. Applications of the Swiss ADME web tool has recently contributed greatly to the design and development of anticancer, tubercular, and antimicrobial agents^33,40,41^. The bioavailability radar demonstrated six physicochemical indices such as lipophilicity (XLOGP3), size, polarity, solubility, saturation, and flexibility (Table 3; Fig. 10). The biophysical range is marked in a pink area which indicates drug-like nature. In the radar image, compounds **1** and **3** are within the limits, which is great but **2** and **4** are slightly above the saturation limit. The blue dots show that, all the compounds in egg spots lead to their efficient efflux by P-glycoprotein (Pgp +) and this confirms that these compounds easily cross the BBB. Furthermore, the skin permeability coefficient (log Kp) was calculated, which appeared to be related to lipophilicity and molecular size. A very negative value of the log Kp estimated for all compounds (− 5.26 to − 6.12 cm/s) indicates low skin penetration.

**Table 3:** Physicochemical properties of compounds (**1**–**4**).

Physicochemical parameters	Dimeric pyridinium bromide (DPB)			
	DPB**1**	DPB**2**	DPB**3**	DPB**4**
Lipophilicity (XLOGP3)	3.41	3.96	2.71	3.72
Size (MW; g/mol)	256.39	290.40	286.42	320.43
Molar reactivity	82.30	92.53	91.11	101.34
Polarity (TPSA; Å^2^)	7.76	7.76	59.80	59.80
Solubility (LogS)	− 3.65	− 4.48	− 3.35	− 4.18
Saturation (Fraction; Csp3)	0.41	0.20	0.41	0.20
Flexibility (number of rotatable bonds)	6	4	6	4
HBA(N + O)	0	0	0	0
HBD(NH + OH)	0	0	2	2
BBB permeant	Yes	Yes	Yes	Yes
Log Kp (skin permeation; cm/s)	− 5.44	− 5.26	− 6.12	− 5.93
P-gp substrate	Yes	Yes	Yes	Yes
Log Po/w	2.75	2.98	1.93	2.16
Bioavailability score	0.55	0.55	0.55	0.55
CYP1A2, CYP2C19, CYP2C9, CYP2D6, and CYP3A4	CYP2D6 (yes)	CYP2D6 (yes)	CYP2D6 (yes)	CYP2D6 (yes)

In Table 3 values confirm that all compounds (**1**–**4**) follow Lipinski's five rules, as a result of which these compounds are expected to have excellent drug-like properties. The fact that these new compounds have molecular weights below 500 indicates that these compounds are easier to transport, diffuse and absorb than larger molecules. Compounds **1** and **2** show no hydrogen bond acceptors (nHA) whereas **3** and **4** show two hydrogen bond acceptors and none of the donor (nHD) atoms in these compounds, which are less than 10 and 5, respectively (Table 4; Fig. 10).

**Table 4:** Binding interaction of compounds with EGFR (1M17).

S. no.	Dimeric pyridinium bromide (DPB)	Binding energy score
1	DPB **1**	− 6.82
2	DPB **2**	− 6.91
3	DPB **3**	− 5.14
4	DPB **4**	− 5.60

The number of rotatable bonds in these compounds ranges from 4 to 6 which is within the range. The TPSA values of compounds **1** and **2** were 7.76 and 3 and 4 showed the same value of 59.80, which is below the normal range and can easily cross the blood–brain barrier. From the above description, the compounds are within the parameter range of Lipinski's five rules (i) molecular weight ≤ 500 Da, (ii) LogP < 5, (iii) nHBD ≤ 5, (iv) nHBA ≤ 10, (v) TPSA < 140 Å^2^, which leads to adherence to criteria for oral drugs.

### Targeting epidermal growth factor receptor

Epidermal growth factor receptor (EGFR) is a protein found on the surface of cells^42^. It plays an important role in cell proliferation and is often targeted by anticancer drugs^43^. EGFR has been especially studied in breast cancer, as it is an important target for the development of new treatment^44^. Research has shown that blocking EGFR can lead to decreased tumor growth and improved survival rates^45^. Many current therapies are designed to specifically target EGFR, making it a key focus for cancer research. Targeting EGFR is important for this study as dimeric pyridinium bromides show potent activity against breast cancer cells.

**Figure 11:**
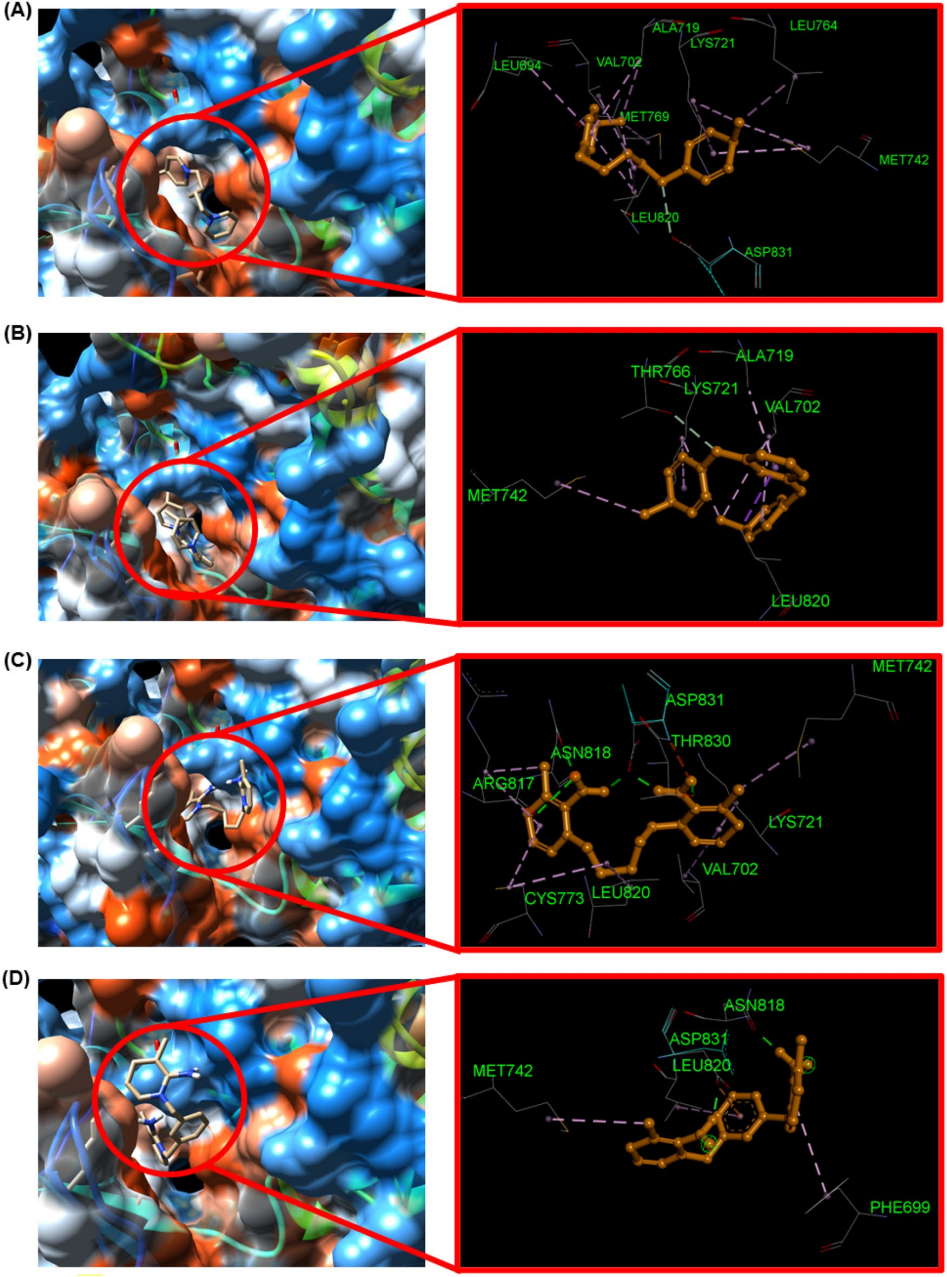
The best docking poses and interactions of compounds (**1**–**4**, a–b) with EGFR.

The newly synthesized compounds were docked with EGFR, and their binding affinity and interactions were shown in Fig. 11. All four compounds interact strongly with EGFR (Table 4). 1,1′Pentane-1,5-diyl)bis(4-methylpyridin-1-ium)bromide **1** showed a carbon–hydrogen bond (C–H) interaction formed between the carbon atom of the CH_2_ moiety and ASP831 with a distance of 3.05 Å (Fig. 11). This compound also formed 8 alkyl interactions with EGFR, with the first two interactions between the methyl moiety and LEU764 and MET769 with distances of 3.98 and 4.37 Å, respectively (Fig. 11). The second four interactions are formed between the tolyl moiety and LYS721, MET742, LEU694, and LEU820 with distances of 5.19, 5.25, 5.21, and 4.67 Å, respectively (Fig. 11). The last two interactions are formed between the CH_2_ moiety and VAL702 and LEU820 with distances of 4.84 and 4.88 Å, respectively (Fig. 11). Furthermore, 1,1'pentane -1,5-diyl)bis(4-methylpyridin-1-ium)bromide **1** stabilizes the interaction of ASP831, THR830, GLU738, GLY772, LEU768, and THR766 with EGFR via vander Waals interactions.

1,1′(1,3-Phenylenebis(methylene))bis(4-methylpyridinum)bromide **2** showed a carbon–hydrogen bond (C–H) interaction formed between the carbon atom of the CH_2_ moiety and THR766 with a distance of 2.90 Å (Fig. 11). Also, showed one π–σ interaction between the phenyl moiety and LEU820 with a distance of 3.55 Å and one π–alkyl interaction between the phenyl moiety and ALA719 with a distance of 4.08 Å (Fig. 11). 1,1′(1,3-Phenylenebis(methylene))bis(4-methylpyridinum)bromide **2** formed 4 alkyl interactions with EGFR, with the first two interactions between the methyl moiety and MET742 and LYS721 with distances of 4.82 and 5.13 Å, respectively (Fig. 11). The second two interactions are formed between the tolyl moiety and LYS721 and VAL702 with distances of 4.98 and 4.67 Å, respectively (Fig. 11). Furthermore, compound **2** stabilizes the interaction of GLN767, THR766, LEU768, MET769, LY772, LEU694, PHE699, ASP831, THR830, PHE832 and GLU738 with EGFR via van der Waals interactions.

1,1′(Pentane-1,5-diyl)bis(2-amino-3-methylpyridin-1-ium)bromide **3** showed four hydrogen bonding interactions, the first formed between the hydrogen atom of the NH_2_ substituent and ARG817 with a distance of 2.89 Å, the second formed between the hydrogen atom of the NH_2_ substituent and ASN818 with a distance of 1.84 Å, the third formed between the hydrogen atom of the NH_2_ substituent and THR830 with a distance of 2.03 Å and the fourth formed between the hydrogen atom of the NH_2_ substituent and ASP831 at a distance of 2.54 Å (Fig. 11). 1,1′(Pentane-1,5-diyl)bis(2-amino-3-methylpyridin-1-ium)bromide **3** also formed 6 π-alkyl interactions with EGFR, with the first three interactions between the phenyl moiety and LYS721, VAL702, and CYS773 with distances of 3.86, 4.87, and 4.49 Å, respectively. The second two interactions are formed between the methyl substituent and MET742 and ARG817 with distances of 4.32 and 3.60 Å, respectively. The last interaction formed between the CH_2_ moiety and LEU820 with distances of 4.26 Å (Fig. 11). Furthermore, 1,1′(pentane-1,5-diyl)bis(2-amino-3-methylpyridin-1-ium)bromide**3** stabilizes the interaction of THR766, and GLU738 with EGFR via van der Waals interactions.

1,1′(1,3-Phenylenebis(methylene))bis(-2-amino-3-methylpyridin)bromide **4** showed two hydrogen bonding interactions, the first formed between the hydrogen atom of the NH_2_ substituent and ASN818 with a distance of 1.77 Å and the second formed between the hydrogen atom of the NH_2_ substituent and ASP831 with a distance of 1.78 Å (Fig. 11). Also, showed one π–anion interaction between the phenyl moiety and ASP831 with a distance of 2.78 Å and one π–alkyl interaction between the tolyl moiety and PHE699 with a distance of 4.74 Å (Fig. 11). 1,1'(1,3-Phenylenebis(methylene))bis(-2-amino-3-methylpyridin)bromide **4** formed 2 π-alkyl interactions with EGFR, with the first interaction between the phenyl moiety and PHE699 with distances of 4.74 Å and the second interaction formed between the phenyl moiety and LEU820 with distances of 5.11 Å. Also, one alkyl interaction between the methyl substituent and MET742 with distances of 4.86 Å (Fig. 11). Furthermore, 1,1′(1,3-phenylenebis(methylene))bis(-2-amino-3-methylpyridin)bromide **4** stabilizes the interaction of CYS773, VAL702, ARG817, THR830, LYS721, ASP813, THR766, GLU738 and PHE832 with EGFR via van der Waals interactions.

The observed binding energy score was consistent with the experimental anticancer activity, again testifying to the potent anticancer activity of the compounds.

## Conclusion

We have prepared novel potent breast cancer drug molecules from non-toxic and inexpensive method. Overall atom economy, environmental factor, environmental catalyst and product mass intensity values gives additional merits for this synthetic method. Synthesized flexible dimeric imidazolium bromides showed less toxicity against normal mammary epithelial cells. Novel dimeric pyridinium bromides showed excellent anticancer response against tested breast cancer cell lines. Alkyl substituted flexible dimeric pyridinium bromide **1** and **2** showed potential anticancer response than the amino substituted flexible dimeric pyridinium bromides **3** and** 4**. In cell cycle, novel flexible dimeric pyridinium bromides showed significant arrest in the G2/M phase by nearly three folds, when compared with control drug. We have studied the targeting epidermal growth factor receptor for all the synthesized flexible amino substituted and methyl substituted dimeric pyridinium bromides. In literature, they have used expensive catalyst and involved multi step synthesis to reach their target molecules from expensive starting materials. Whereas, our current breast cancer drug molecules are obtained from easily available inexpensive starting materials. We have not used any expensive catalyst during the preparation and involves single step to reach our target molecules. We have compared the IC_50_ values from available literatures, our target molecules showed least IC_50_ values. So, our simple/substituted dimeric pyridinium bromides showed potent anticancer behaviour against tested breast cancer cell lines.

## Experimental method

### General procedure for the preparation of dimeric pyridinium bromide 1 and 3

1,5-Dibrompentane(1.0equi.) and 4-methyl pyridine/2-amino-3-methylpyridine(2.02 equi.) were dissolved in 80 mL of dry acetonitrile under refluxing condition for 2 h afforded the desired compound**1** and** 3**.

#### 1,1′(Pentane-1,5-diyl)bis(4-methylpyridin-1-ium)bromide 1

Yield: 2.94 g, 96%; Liquid; ^1^H NMR (400 MHz; D_2_O) δ(ppm): 1.44–1.51(quin., J = 8 Hz, 2H); 2.10–2.18 (quin., J = 8 Hz, 4H); 2.70 (s, 6H); 4.62–4.65 (t, J = 8 Hz,4H); 7.95–7.96 (d, J = 4 Hz,4H); 8.75–8.76 (d, J = 4 Hz,4H); ^13^CNMR(100 MHz; D_2_O) δ (ppm): 20.1, 20.6, 28.4, 59.0, 127.3, 141.7, 158.5, HRMS: *m/z* 128.1000; Anal. Calculated for C_17_H_24_N_2_Br_2_: C, 49.06; H, 5.76; N, 6.73; Found: C, 49.02; H, 5.72; N, 6.69 (Supplementary Figures S1–S3).

#### 1,1′(Pentane-1,5-diyl)bis(2-amino-3-methylpyridin-1-ium)bromide 3

Yield: 3.79 g, 92%;Liquid; ^1^H NMR (400 MHz; D_2_O) δ (ppm): 1.37–1.43 (quin., J = 8 Hz, 2H); 1.77–1.84 (quin., J = 8 Hz, 4H); 2.0 (s, 6H); 4.12–4.15 (t, J = 8 Hz, 4H); 6.76–6.80 (t, J = 8 Hz, 2H); 7.63–7.65; (d, J = 8 Hz, 2H); 7.71–7.72 (d, J = 4 Hz, 2H); ^13^C NMR (100 MHz; D_2_O) δ (ppm):16.9, 22.2, 26.4, 54.1, 113.6, 123.7, 137. 4, 141.7, 152.5, HRMS: *m/z*143.1150; Anal. Calculated for C_17_H_26_N_4_Br_2_: C, 45.73; H, 5.82; N, 12.55; Found: C, 45.69; H, 5.78; N, 12.51 (Supplementary Figures S7–S9).

### General procedure for the preparation of dimeric pyridinium bromide 2, 4

*m*-Xylene dibromide (1.0equi.) and 4-methyl pyridine/2-amino-3-methylpyridine(2.02 equi.) were dissolved in 80 mL of dry acetonitrile at room temperature for 2 h afforded the desired compound **2**,** 4**.

#### 1,1′(1,3-Phenylenebis(methylene))bis(4-methylpyridinum)bromide 2

Yield: 3.78 g, 94%; Liquid; ^1^H NMR (400 MHz; D_2_O) δ (ppm): 2.55 (s, 6H); 5.68 (s, 4H); 7.78–7.80 (d, J = 8 Hz, 2H); 7.45–7.44 (d, J = 4 Hz, 2H); 7.25–7.30 (s, 1H); 7.15–7.18 (t, J = 8 Hz, 1H); 8.61–8.62 (d, J = 4 Hz, 4H); 7.80–7.77 (d, J = 4 Hz, 4H); ^13^C NMR (100 MHz; D_2_O) δ (ppm): 29.4, 63.0, 143.2, 134.3, 130.6, 130.0, 129.6, 129.1, 128.7, HRMS: *m/z*145.1100; Anal. Calculated for C_20_H_22_N_2_Br_2_: C, 53.33; H, 4.88; N, 6.22; Found: C, 53.29; H, 4.84; N, 6.18 (Supplementary Figures S4–S6).

#### 1,1′(1,3-Phenylenebis(methylene))bis(-2-amino-3-methylpyridin)bromide 4

Yield: 3.46 g, 95%; mp.: 74–76 °C; ^1^HNMR (400 MHZ; D_2_O) δ (ppm): 4.70 (s, 6H); 5.42 (s, 4H); 6.17 (s, 1H); 6.84–6.87 (t, J = 8 Hz, 2H); 7.27–7.29 (d, J = 8 Hz, 2H); 7.44–7.48 (t, J = 8 Hz, 1H); 7.74–7.76 (d, J = 8 Hz, 2H); 7.70–7.72 (d, J = 8 Hz, 2H); ^13^C NMR (100 MHz; D_2_O) δ (ppm): 16.6, 56.4, 113.8, 122.4, 124.1, 127.3, 130.1, 133.2, 137.6, 142.3, 153.1, HRMS: *m/z* 160.1200; Anal. Calculated for C_20_H_24_N_4_Br_2_: C, 50.02; H, 5.04; N, 11.66; Found: C, 49.98; H, 4.99; N, 11.63 (Supplementary Figures S10–S12).

### Cell culture

The human breast cancer cell line, MCF7 (ATCC: HTB-22™), MDA-MB-231 (ATCC: CRM-HTB-26™), T-47D (ATCC: HTB-133™), and MCF 10A (ATCC: CRL-10317™) were purchased from the American type culture collection (Rockville, MD).

### MTT assay

5 × 10^3^ cells are seeded in a 96-well plate and incubated for growth under the conditions mentioned above. Once confluency is reached, the cells are treated with different concentrations of simple/amino substituted dimeric pyridinium bromides **1**–**4** and incubated for 24 h. After incubation, the media is removed, and 20 μL of MTT is added and incubated for further 3 h. After 3 h, MTT is removed, and DMSO is added to dissolve the formazan crystals. The absorbance is measured at 570 nm, and the percentage of cell death is calculated using the formula

$$\% {\text{ of}}\;{\text{ cell }}\;{\text{death }} = {\text{ Absorbance }}\;{\text{of}}\;{\text{ control }} - {\text{ Absorbance }}\;{\text{of }}\;{\text{treated }}/{\text{Absorbance }}\;{\text{of}}\;{\text{ control }} \times {1}00$$

### Ethical approval

This article does not contain any studies with human participants or animals performed by the authors.

### Consent to participate

We comply with the ethical standards. We provide our consent to take part.

### Supplementary Information

Supplementary Figures.

## Data Availability

The datasets used and/or analyzed during the current study available from the corresponding author on reasonable request.
