# Supplementary material for: Synthesis of potent MDA-MB 231 breast cancer drug molecules from single step
Source: Sci Rep. 2023 Oct 25;13:18241. doi: 10.1038/s41598-023-45455-y (PMC10600176; doi:10.1038/s41598-023-45455-y)
Supplement: Supplementary file 1 — Supplementary Figures. [file 41598_2023_45455_MOESM1_ESM.docx]

**Synthesis of potent MDA-MB 231 breast cancer drug molecules from single step**

**Senthilnathan Govindaraj^a^, Kilivelu Ganesan^a*^, Mahendiran Dharmasivam^b^, Lakshmisundaram Raman^c^, Mohammad Mujahid Alam^d^, Mohammad Amanullah^e^**

^a^*PG& Research Department of Chemistry, Presidency College, Chennai – 600005, India.*

*^b^Centre for Cancer Cell Biology and Drug Discovery, Griffith Institute for Drug Discovery, Griffith University, Nathan, Brisbane, Queensland 4111, Australia*

*^c^Sri Ramachandra Faculty of Pharmacy, Sri Ramachandra Institute of Higher Educational and Research (DU), Porur, Chennai 600116, India.*

*^d^Department of Chemistry, College of Science, King Khalid University, PO Box 9004, Abha 61413, Kingdom of Saudi Arabia*

*^e^Department of Clinical Biochemistry, College of Medicine, King Khalid University, Abha 61413, Kingdom of Saudi Arabia*

**SUPPORTING INFORMATION**

1. ^1^H NMR Spectrum of compound  **1** Figure S1
2. ^13^C NMR Spectrum of compound **1** Figure S2
3. HRMS spectrum of compound **1** Figure S3
4. ^1^H NMR Spectrum of compound  **2** Figure S4
5. ^13^C NMR Spectrum of compound  **2** Figure S5
6. HRMS spectrum of compound **2** Figure S6
7. ^1^H NMR Spectrum of compound  **3** Figure S7
8. ^13^C NMR Spectrum of compound  **3** Figure S8
9. HRMS spectrum of compound **3** Figure S9
10. ^1^H NMR Spectrum of compound  **4** Figure S10
11. ^13^C NMR Spectrum of compound  **4** Figure S11
12. HRMS spectrum of compound **4** Figure S12

1,1' ( Pentane -1,5-diyl)bis(4-methylpyridin-1-ium)bromide **1**

Figure S1

1,1'( Pentane -1,5-diyl)bis(4-methylpyridin-1-ium)bromide **1**

Figure S2

1,1'( Pentane -1,5-diyl)bis(4-methylpyridin-1-ium)bromide **1**


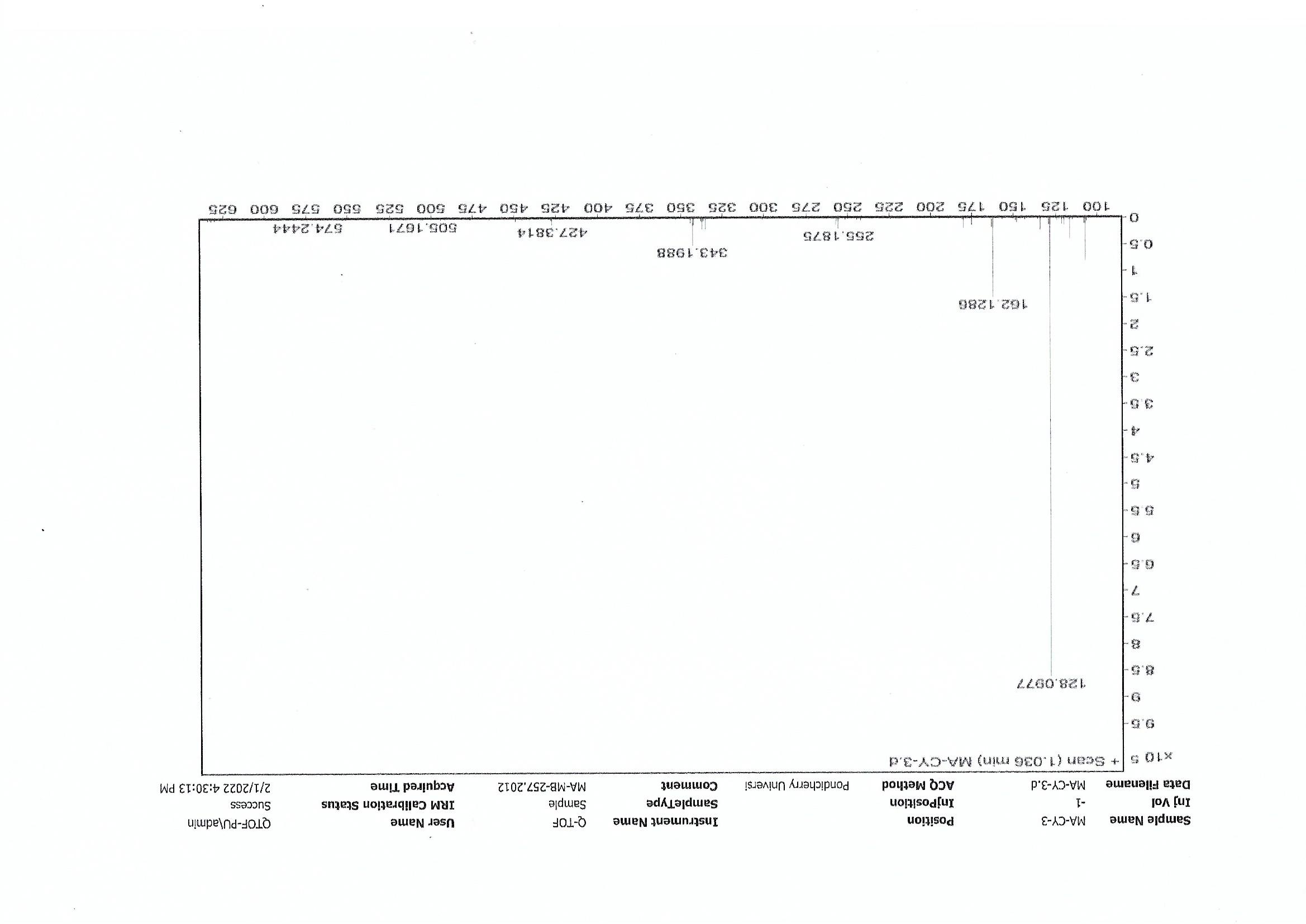


Figure S3

1,1'( 1,3-Phenylenebis(methylene))bis(4-methylpyridinum)bromide **2**


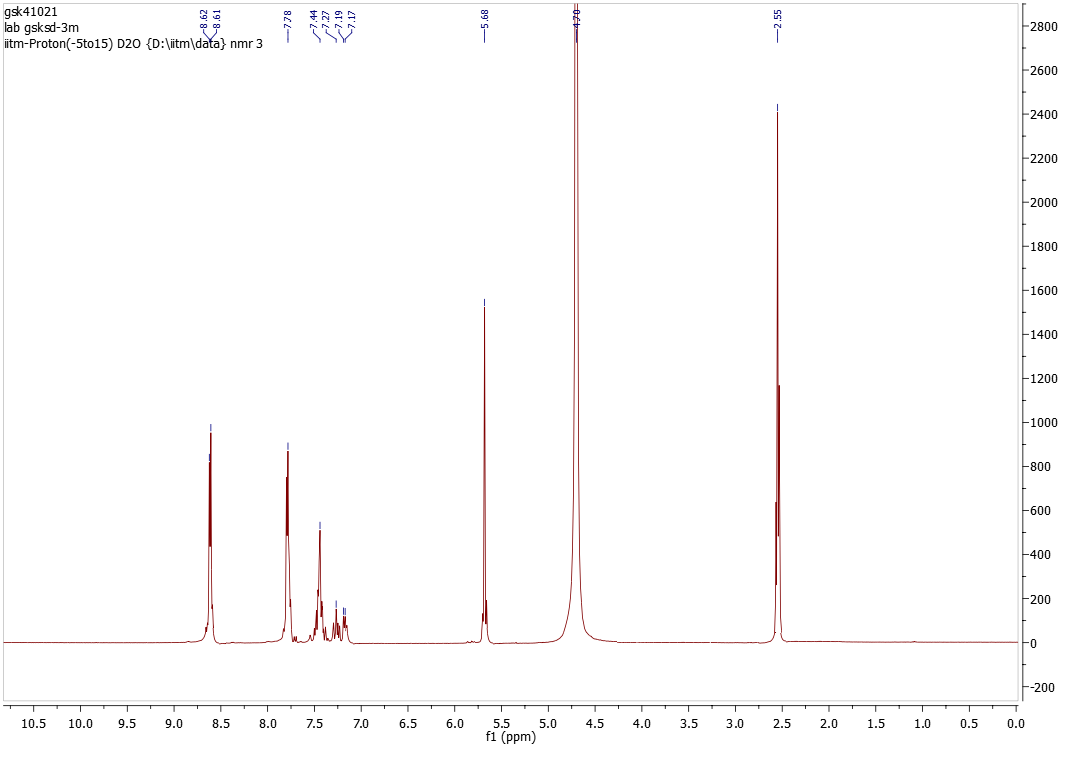


Figure S4

1,1' (1,3-Phenylenebis(methylene))bis(4-methylpyridinum)bromide **2**


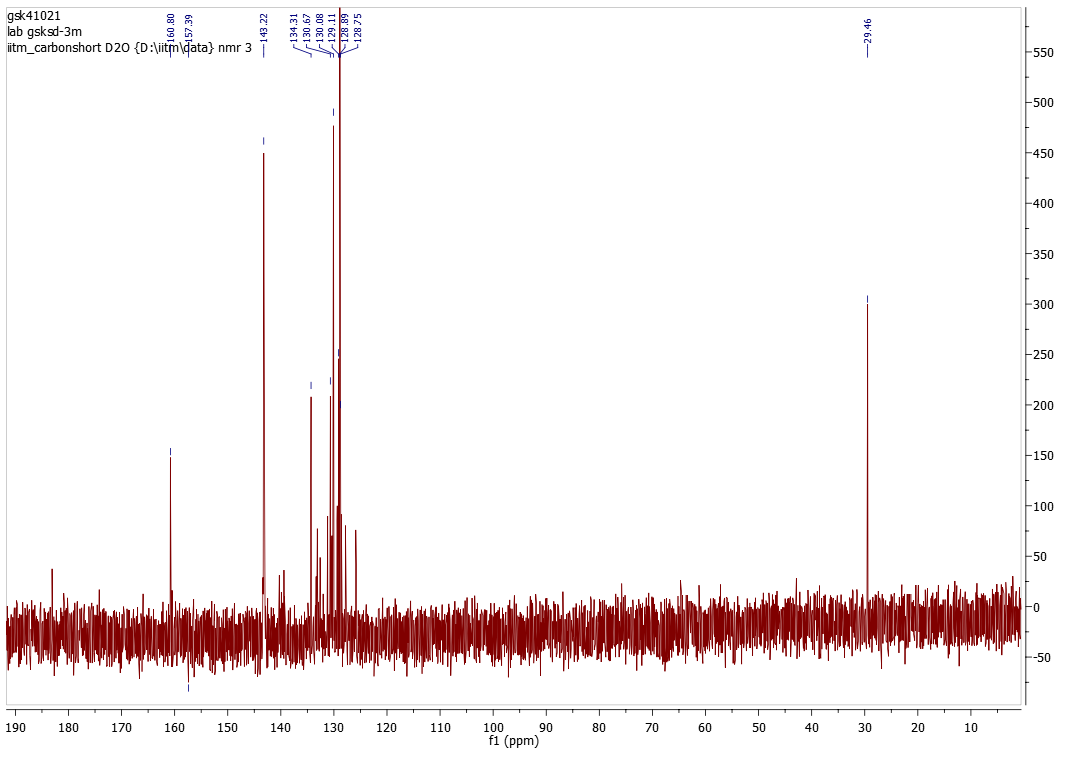


Figure S5

1,1'(1,3-Phenylenebis(methylene))bis(4-methylpyridinum)bromide **2**


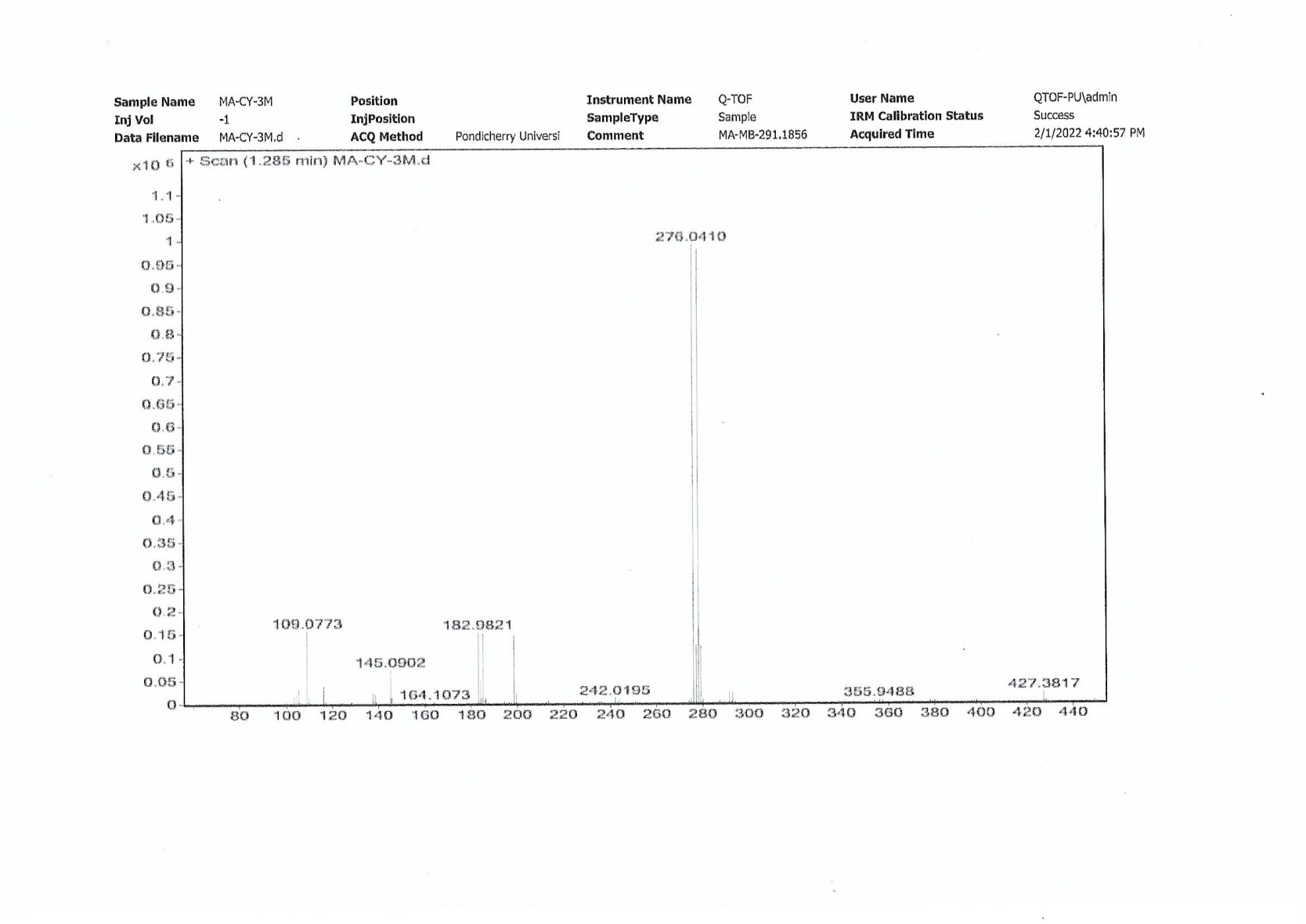


Figure S6

1,1 '( Pentane-1,5-diyl)bis(2-amino-3-methylpyridin-1-ium)bromide **3**


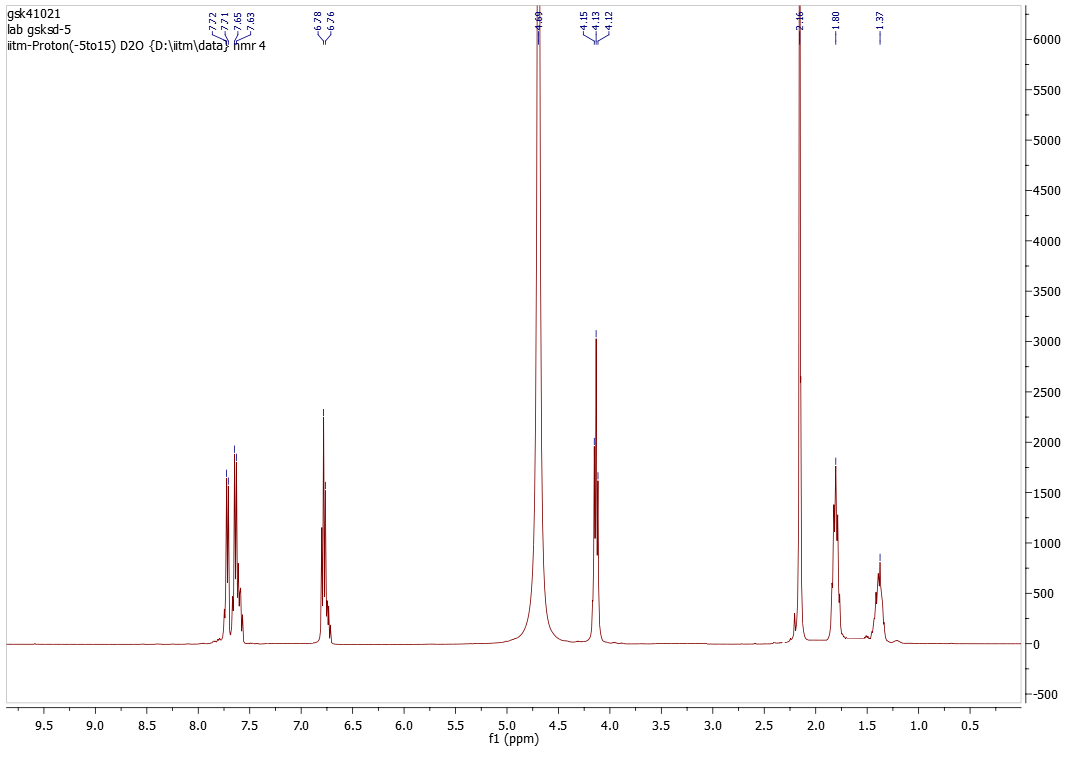


Figure S7

1,1'( Pentane-1,5-diyl)bis(2-amino-3-methylpyridin-1-ium)bromide **3**


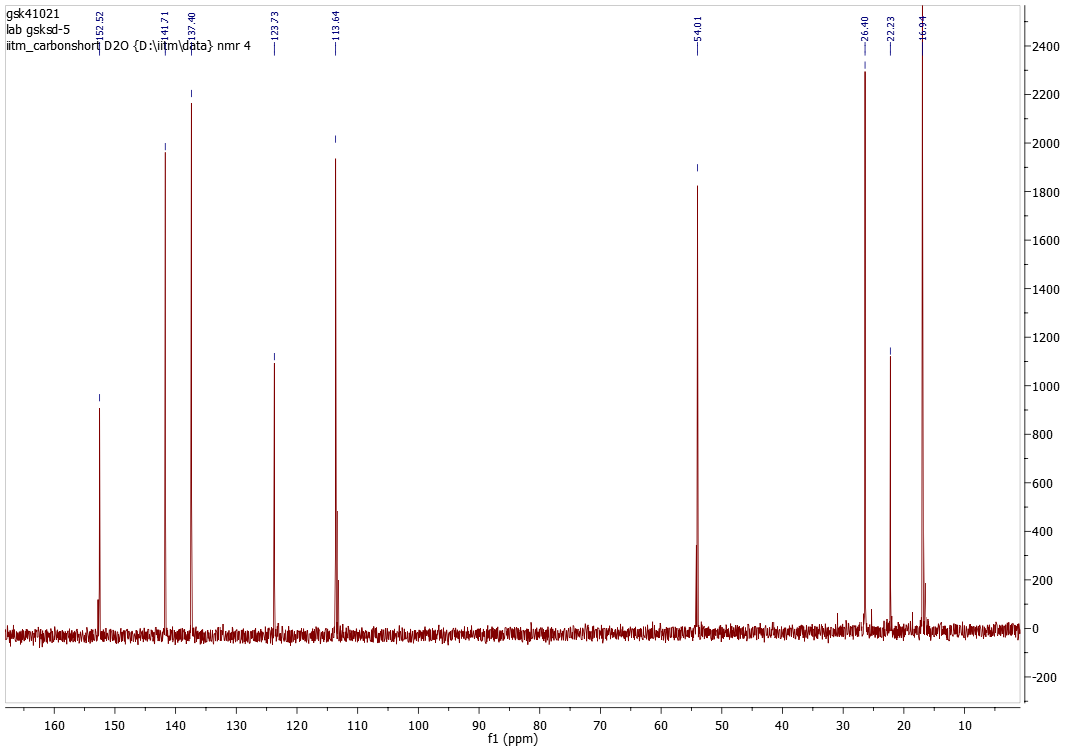


Figure S8

1,1'( Pentane-1,5-diyl)bis(2-amino-3-methylpyridin-1-ium)bromide **3**


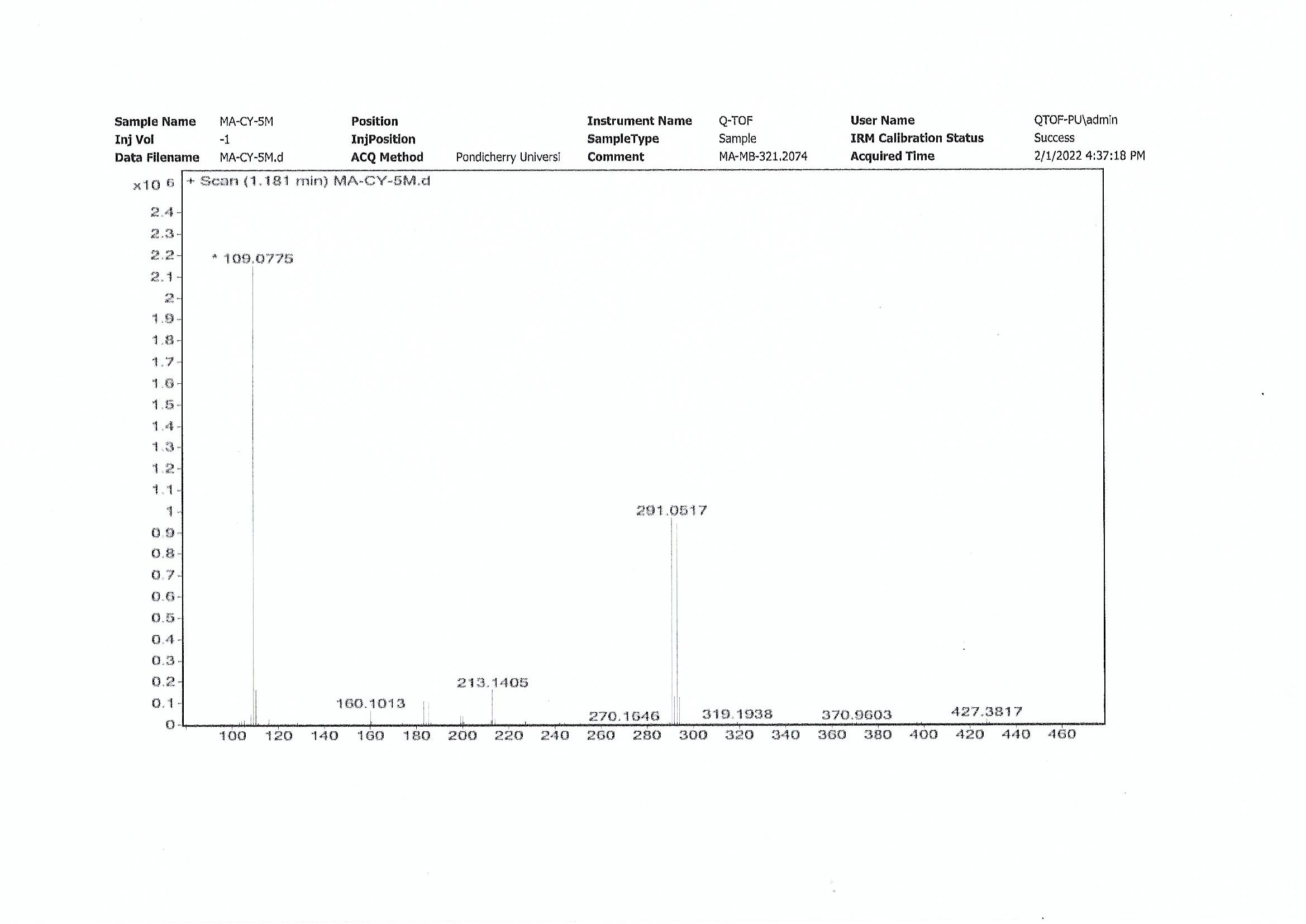


Figure S9

1,1'(1,3-Phenylenebis(methylene))bis(3-methyl-1λ^4^pyridin-2-amin),bromide **4**

Figure S10

1,1'(1,3-Phenylenebis(methylene))bis(3-methyl-1λ^4^pyridin-2-amin),bromide **4**

Figure S11

1,1'(1,3-Phenylenebis(methylene))bis(3-methyl-1λ^4^pyridin-2-amin),bromide **4**


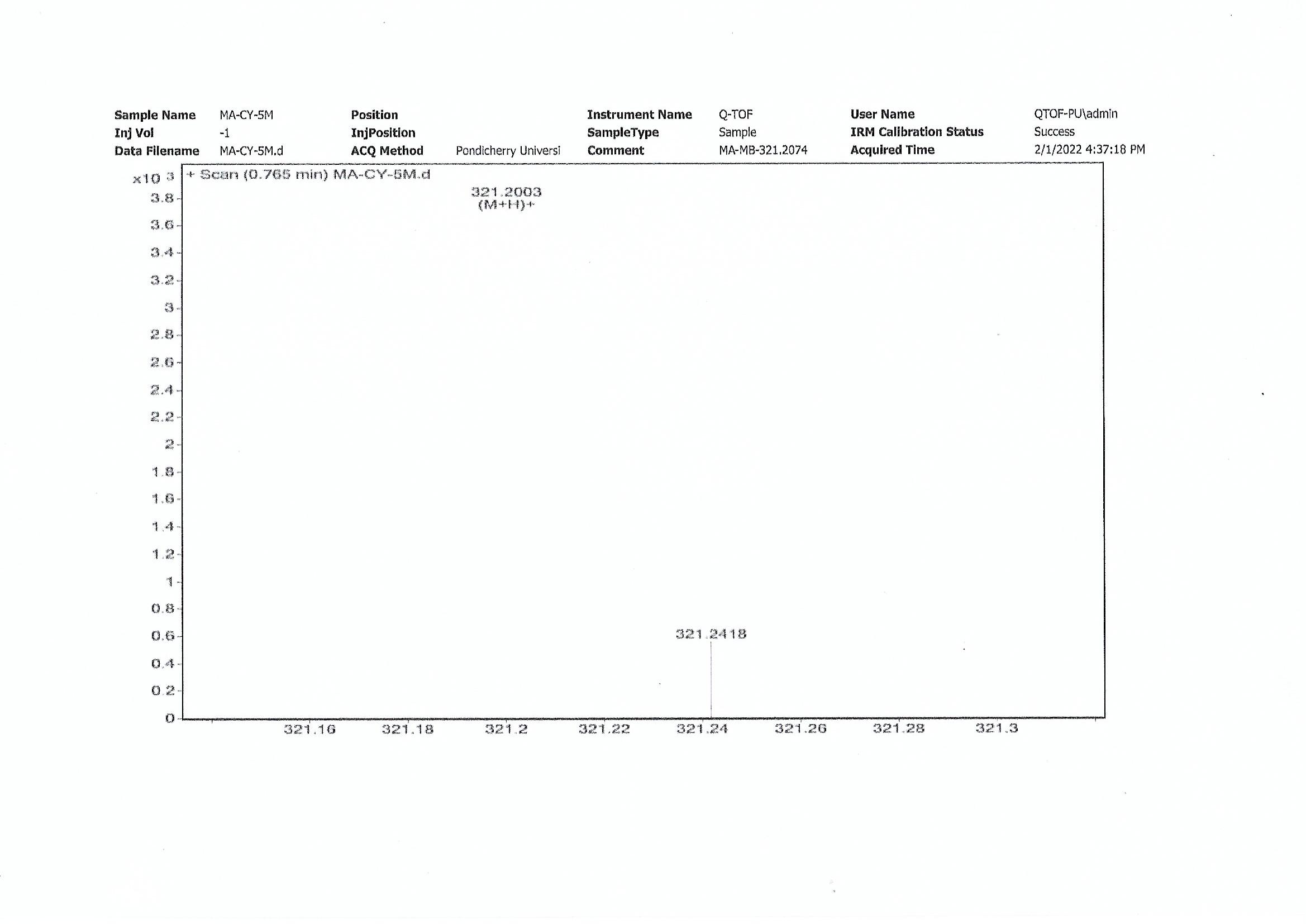


Figure S12
